# Alteration of Behavioral Inhibitory Control in High-Altitude Immigrants

**DOI:** 10.3389/fnbeh.2021.712278

**Published:** 2021-12-10

**Authors:** Jiazheng Wang, Liqin Zheng, Zedong Wang, Xiao Wu, Ning Ma, Tao Zhang, Kai Chen, Bharat B. Biswal, Qun Yang, Hailin Ma

**Affiliations:** ^1^MOE Key Laboratory for Neuroinformation, Center for Information in Medicine, School of Life Science and Technology, The Clinical Hospital of Chengdu Brain Science Institute, University of Electronic Science and Technology of China, Chengdu, China; ^2^Key Laboratory of Brain, Cognition and Education Sciences, Ministry of Education, School of Psychology, South China Normal University, Guangzhou, China; ^3^Center for Mental Health Development and Research, Xihua University, Chengdu, China; ^4^Department of Clinical Psychology, Fourth Military Medical University, Xi’an, China; ^5^Plateau Brain Science Research Center, Tibet University, Lhasa, China; ^6^Plateau Brain Science Research Center, South China Normal University, Guangzhou, China

**Keywords:** high-altitude, behavioral inhibitory control (BIC), two-choice oddball task, time-frequency analysis, delta, theta

## Abstract

Behavioral inhibitory control (BIC) acts as a key cognitive ability, which is essential for humans to withhold inappropriate behaviors. Meanwhile, many studies reported that long-term exposure to high altitude (HA) may affect cognitive ability. However, it is not clear whether long-term exposure to HAs may affect the BIC of an individual. To clarify the role of altitude in the behavioral control of adults and the underlying neural mechanism, we explored the BIC neural activity profiles of healthy immigrants from low-altitude (LA) regions to HA regions. Combining a two-choice oddball paradigm and electrophysiological techniques, this study monitored the N2 and P3 event-related components and neural oscillations across LA and HA groups. Results showed longer reaction times (RTs) for the HA group than the LA group. Relative to the LA group, lower N2 and P3 amplitudes were observed for the HA group. Significant positive correlations were also found between P3 amplitude and theta/delta band power across both groups. Importantly, lower theta/delta band powers were only observed for the HA group under the deviant condition. Collectively, these findings suggest that long-term exposure to HAs may attenuate BIC during the response inhibition stage and provide valuable insights into the neurocognitive implications of environmental altitude on BIC.

## Introduction

The importance of understanding the influences of high-altitude (HA) exposure on the cognition of human beings has recently gained more attention (Penaloza and Arias-Stella, 2007; Wang et al., 2015). HA exposure is typically characterized by living in a region with lower atmospheric pressure and lower oxygen levels (Singh et al., 2003). Hypoxia (which starts at heights over 2,500 m above sea level) is considered the largest impact of HA living (Virués-Ortega et al., 2006; Bucuk et al., 2008). In relation to cognitive functioning, researchers have widely reported long-term impairments in memory, attention, and executive functioning for immigrants exposed to HAs for a long period of time.

It is well known that executive functioning [i.e., behavioral inhibitory control (BIC)] is one of the most essential cognitive mechanisms governing human behavior, playing a vital role in ideological coordination and behavioral self-regulation (Carlson, 2005). In fact, BIC is a critical component of executive functioning, affecting the ability of a person to suppress or delay dominant reactions to proactively achieve certain goals. Declines in BIC have many significant negative influences on daily life, including impulsive behaviors and cognitive deficits (Carlson and Moses, 2001; Liddle et al., 2001; Simmonds et al., 2008; Ames et al., 2014; Morein-Zamir et al., 2014; Prisciandaro et al., 2014). Neuroimaging evidence shows reduced activity in the anterior cingulate cortex (ACC) and prefrontal cortex activation in children with behavioral control disorders, suggesting that BIC disruptions are related to atypical brain activity signatures (Stadler et al., 2007). Divergent brain activity patterns and structure have also been reported for individuals with HA exposure (Yan et al., 2011a,b). Immigrants from HAs show decreased activation of the left middle occipital gyrus (Yan et al., 2011a,b) as well as significantly decreased gray matter volumes in inferior and middle frontal gyrus and ACC (Zhang et al., 2013) relative to low-altitude (LA) residents, regions commonly associated with BIC (Liddle et al., 2001; Simmonds et al., 2008). These findings have led researchers to propose that such atypicalities in the prefrontal cortex and ACC may consequently impact the behavior of individuals living in HA areas. In a similar vein, the P3 event-related potential (ERP) component – a well-known neural marker of attention resources and behavioral inhibition (Roberts et al., 1994; Liotti et al., 2000; Bokura et al., 2001; Sehlmeyer et al., 2010; Wang et al., 2015) – also shows reduced amplitudes in high-amplitude residents (Wang et al., 2015; Hailin et al., 2018; Ma et al., 2019). The ERP technique also has a distinct temporal advantage over other neuroimaging methods (e.g., fMRI), reflecting cortical variations with millisecond resolution.

As a whole, it seems that divergent brain activity may be related to abnormal behavior in HA immigrants. To clarify this issue in relation to BIC, this study seeks to determine whether long-term exposure to HAs affects BIC at the behavioral and neural levels. To this end, the two-choice oddball task provides a convenient means of evaluating this research. To date, the Go/No-Go and stop-signal tasks have been used as common measures of BIC (Goldstein et al., 2007; Cooper and Hughes, 2017); however, in both of these paradigms, participants do not make any responses when novel (target) stimuli appear, thereby making BIC reaction times (RTs) virtually unobservable. In contrast, the two-choice oddball task overcomes this limitation by having participants respond to both standard (85% probability) and deviant (15% probability) cases by pressing different keys (Yuan et al., 2008, 2012; Wang et al., 2011). Since the standard stimulus is presented much more frequently than the deviant stimulus, participants have to inhibit prepotent responses to the standard stimulus during deviant trials. This paradigm also supplies BIC-specific ERPs (N2 and P3) which are free of motor contamination due to counterbalanced responses to standard and deviant stimuli (Yuan et al., 2008, 2012; Wang et al., 2011). The two-choice oddball task and ERP technique have revealed that BIC contains complex procedures (e.g., conflict monitoring and subsequent response inhibition), reflected by frontocentral N2 and P3 components (Yuan et al., 2008, 2012, 2020; Wang et al., 2011). However, it is important to consider that changes in stimulus presentation alter potentials and frequency power variation in addition to alterations in brain activation (Chen et al., 2008; Cavanagh and Cohen, 2009; Cavanagh et al., 2010; Womelsdorf et al., 2010; Anguera et al., 2013). In fact, theta/delta band power can explain a significant proportion of P3 variance in oddball and Go/No-Go tasks investigating BIC (Polich, 2007; Ergen et al., 2008; Harper et al., 2014; Bachman and Bernat, 2018). Prefrontal delta and theta bands are more sensitive to No-Go stimuli (which draw on the response inhibition stage of BIC) than to Go stimuli (Harper et al., 2014). The stop-signal task has also revealed that prefrontal theta band power and increased coherence only occur during low-goal-conflict trials, suggesting that these metrics can also be used to index response inhibition (De Blasio and Barry, 2018).

The ERP techniques have been used to extensively explore the time course and neurocognitive mechanisms of BIC during physical and cognitive activities (Wang et al., 2011; Yuan et al., 2012). Adding to this body of literature and exploring the modulation of BIC across HA and LA, this study implements a two-choice oddball paradigm with co-registered ERP recordings in immigrants living in HA regions and sea-level residents. BIC was expected to be influenced by altitude, indexed by lower N2 and P3 amplitudes and weaker theta/delta band power for HA residents.

## Materials and Methods

### Participants

We used power analysis to calculate the number of participants required in our study using G power software (Version 3.1.9.7). The result showed that the sample size of each group should be 26 (*d* = 0.8, α = 0.05, 1 - β = 0.8). A total of 71 Han college students (all right-handed) took part in this study and signed an informed consent letter before the experiment. All participants were born and raised at the sea level, with 37 individuals currently living in HA (3,680 m) regions for at least 3 years designating the HA group: 18 males, 21.08 ± 1.19 years, and the remaining 34 participants never having lived in a HA area are designated as the LA group: 15 males, 20.76 ± 1.05 years. All participants reported standard visual acuity or corrected vision (glasses or contacts) and did not have a history of mental disorder or familial-hereditary disease. HA and LA groups were matched for age [*F*(1, 69) = 1.136, *p* > 0.005], educational level [*F*(1, 69) = 0.081, *p* > 0.05], gender [*F*(1, 69) = 0.266, *p* > 0.05], and intelligence (Raven’s standard intelligence scale [*F*(1, 69) = 2.965, *p* > 0.05]. Before the experiment, all the students signed an informed consent letter, and the Ethics Committee approved the test in Tibet University, XZDXLL2017008.

### Stimuli and Procedure

This study implements a two-choice oddball task consisting of a practice phase and two experimental blocks. Before the formal experiment, participants completed 10 practice trials to familiarize themselves with the procedure; all participants had to achieve 100% accuracy before conducting the main experiment. Each experimental block contained 200 trials (170 standard trials and 30 deviant trials) with the uppercase letters “W” and “M” representing the standard and deviant trials, respectively. Stimuli were randomized across participants.

**FIGURE 1:**
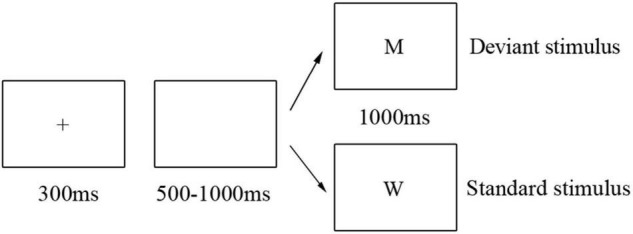
Materials and procedure. Trial progression for the two-choice oddball paradigm. Deviant stimuli (15% probability) were indicated by the letter “M,” and standard trials (85% probability) were indicated by the letter “W.”

Participants were tested individually in a dimly lit experimental environment, seated nearly 70 cm from a 17′ LCD monitor. Black letter stimuli subtending approximately 6° × 6° of visual angle were presented on a white background. Each trial began with a 300 ms small black cross, followed by a 500–1,000 ms blank screen. Then, a letter stimulus was presented for 1,000 ms, with participants being required to respond with a binary button press within 1,000 ms (Figure 1). Half of the participants in each group was instructed to press the “F” keyboard button for standard trials and the “J” button on deviant trials as quickly and accurately as possible, with button response reversed for the remaining half of the participants to control potential influences of response hands on ERP responses. Participants were provided with their two-block accuracy at the end of the experiment.

### Event-Related Potential Recording and Analysis

#### Recording

Ag/AgCl electrodes mounted in an elastic cap (Neuroscan Inc., Charlotte, NC, United States) were used to record electroencephalographic (EEG) data across 64 scalp sites (10/20 system, extended). Electrodes were in-line referenced to a reference site located in between Cz and CPz locations, and electrode impedances remained under 5 kΩ. Vertical electrooculograms (EOG) were recorded from above and below the left eye, and horizontal EOG were measured at the outer canthi of both eyes. EEG and EOG data were continuously monitored at a sampling rate of 500 Hz.

#### Preprocessing

The EEG activity of correct trials for each condition was averaged and re-referenced offline in reference to the average mastoid (M1 and M2) values. EEG waveforms were digitally filtered (0.1–30 Hz) and time-locked to stimulus onset of stimuli with a 200 ms pre-stimulus baseline and extending 1,000 ms after stimulus presentation. Eye artifacts were removed using a regression-based approach in Neuroscan software (Version 4.5), and epochs contaminated by other artifacts ±100 μV (e.g., blinking, body movement, and muscle activity) were discarded by automatic artifact rejection. Nine electrode sites (i.e., Fz, F3, F4, FCz, FC3, FC4, Cz, C3, and C4) were selected for statistical analysis. The amplitudes manifested by the N2 (230–330 ms) and P3 components (370–470 ms) were analyzed across conditions, which were theta (4–8 Hz, 300–500 ms) and delta (0.1–3 Hz, 350–550 ms) power bands. The time windows were chosen at the strongest power and highest amplitude time series.

Time-frequency representations (TFRs) of power were computed for EEG segments, time-locked to the 1,000 ms epoch with a sliding 400 ms time window in 20 ms steps. Then, data were multiplied with a Hanning taper and a short-time Fourier transform. Averaged TFRs were calculated for each condition, and the data were normalized to mean power over the baseline interval (-200 to 0 ms) through decibel transform (Delorme and Makeig, 2004; Delorme et al., 2007). These computations were performed using the FieldTrip toolbox of MATLAB (Oostenveld et al., 2011).

### Statistical Analysis

Only correct trials with response times between 200 and 1,000 ms trials were analyzed in this study. In the HA group, we have discarded 477 trials totally (standard condition = 374 and deviant condition = 103), and in the LA group, we have discarded 503 trials totally (standard condition = 391 and deviant condition = 112). Data were exported to STATISTICA, and 2 (Stimulus Type: standard and deviant) × 2 (Group: HA and LA) mixed-method ANOVAs were performed with ERP amplitudes and inter-trial coherence values for all nine channels, frequency bands, and time windows. Then, the regression analysis was implemented to assess whether TFRs accounted for the N2 and P3 time domains. The degrees of the *F*-ratio freedom were corrected using the method of the Greenhouse-Geisser in all analyses.

## Results

### Behavioral Results

The 2 × 2 ANOVA on accuracy responses revealed a significant main effect of the Stimulus Type [*F*(1, 69) = 38.123, *p* < 0.001, η^2^ = 0.356], with accuracy being lower in the deviant condition relative to the standard condition. The main effect of Group was also significant [*F*(1, 69) = 4.460, *p* < 0.050, η^2^ = 0.061], with the HA group yielding lower accuracy than the LA group (Table 1). The interaction was not significant (*p* ≥ 0.05).

**TABLE 1:** Averaged reaction times (RTs) and accuracy values for standard and deviant conditions for low-altitude (LA) and high-altitude (HA) groups (*M* ± *SD*).

	Standard RT	Deviant RT	Standard ACC	Deviant ACC
HA	393.29 ± 5.70	528.83 ± 6.74	96.59% ± 1.77	84.73% ± 1.57
LA	375.50 ± 5.94	507.24 ± 7.02	99.23% ± 1.85	88.94% ± 1.64

**FIGURE 2:**
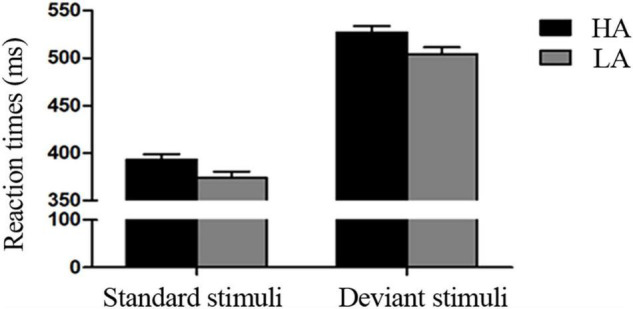
Behavioral results. Mean reaction times (RTs) for low-altitude (LA) and high-altitude (HA) groups under the standard and deviant conditions.

The 2 × 2 ANOVA on RTs showed significant main effects of Stimulus Type [*F*(1, 69) = 1126.710, *p* < 0.001, η^2^ = 0.942] and Group [*F*(1, 69) = 5.323, *p* < 0.050, η^2^ = 0.061]. RTs were longer in the deviant condition than in the standard condition, and the HA group took longer to respond to targets than the LA group (Table 1 and Figure 2). However, the interaction was not significant (*p* ≥ 0.05).

### Event-Related Potential Results

**FIGURE 3:**
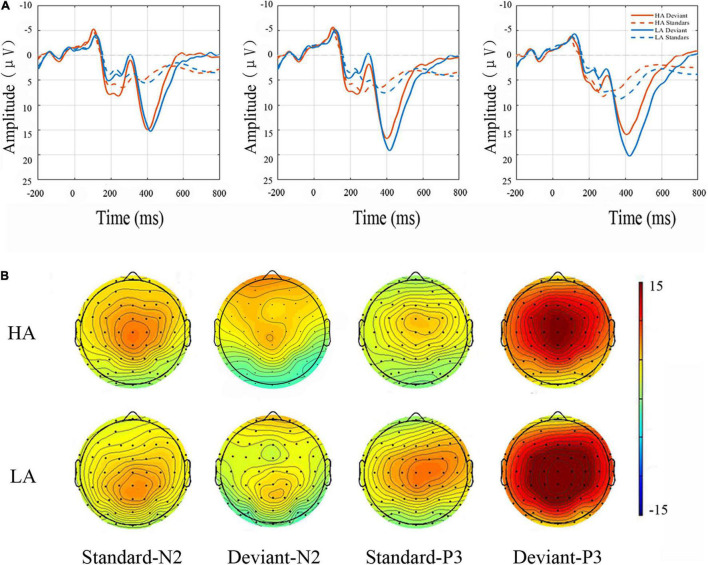
Top panel **(A)** N2 and P3 grand average amplitudes. Event-related potential (ERP) responses elicited to standard and deviant conditions at frontocentral sites (i.e., Fz, FCz, and Cz) for LA and HA groups. Bottom panel: topographic maps **(B)**. Scalp distributions are time-locked to the grand average amplitudes of N2 and P3 components in the standard and deviant conditions.

The main effect of electrode sites was significant for the N2 ERP component [*F*(8, 144) = 9.399, *p* < 0.001, η^2^ = 0.120], with amplitudes being largest at the Fz site (Figure 3). A significant main effect of Group was also found for N2 amplitude [*F*(1, 69) = 4.879, *p* < 0.050, η^2^ = 0.066], with N2 amplitudes being lower in the in HA group than in the LA group. Finally, the deviant condition elicited enhanced N2 amplitudes relative to N2 amplitudes elicited by the standard condition (main effect of the Stimulus Type: *F*(1, 69) = 11.061, *p* < 0.000, η^2^ = 0.138). No interactions were significant for this component (Table 2).

**TABLE 2:** N2 mean amplitudes elicited by standard and deviant conditions at Fz, FCz, and Cz sites in LA and HA groups (*M* ± *SD*).

	Standard-N2		Deviant-N2	
	HA	LA	HA	LA
FZ	5.69 ± 3.74	3.62 ± 3.65	4.61 ± 4.82	2.04 ± 5.54
FCZ	6.74 ± 4.30	4.48 ± 4.23	4.77 ± 5.02	1.66 ± 5.72
CZ	7.48 ± 4.46	6.01 ± 4.29	5.74 ± 5.00	3.78 ± 4.96

As shown in Figure 3, the P3 component was largest at the Cz site [main effect of electrode sites: *F*(8, 251) = 44.697, *p* < 0.001, η^2^ = 0.407] and yielded significant main effects of Group [*F*(1, 68) = 7.611, *p* < 0.050, η^2^ = 0.105] and Stimulus Type [*F*(1, 68) = 285.853, *p* < 0.001, η^2^ = 0.815]. In this study, P3 amplitudes were smaller for participants in the HA group relative to LA residents. The P3 ERP component was also more pronounced for deviant stimuli than for the standard condition (Table 3). No interactions reached significance (*p* ≥ 0.05).

**TABLE 3:** P3 mean amplitudes elicited by standard and deviant conditions at Fz, FCz, and Cz sites in LA and HA residents (*M* ± *SD*).

	Standard-P3		Deviant-P3	
	HA	LA	HA	LA
FZ	3.71 ± 3.19	5.17 ± 3.39	12.26 ± 5.18	14.03 ± 6.59
FCZ	5.01 ± 3.72	6.97 ± 4.14	14.20 ± 5.31	17.72 ± 7.88
CZ	4.65 ± 4.03	7.61 ± 3.94	14.18 ± 5.55	19.16 ± 8.08

### Time-Frequency Results

**FIGURE 4:**
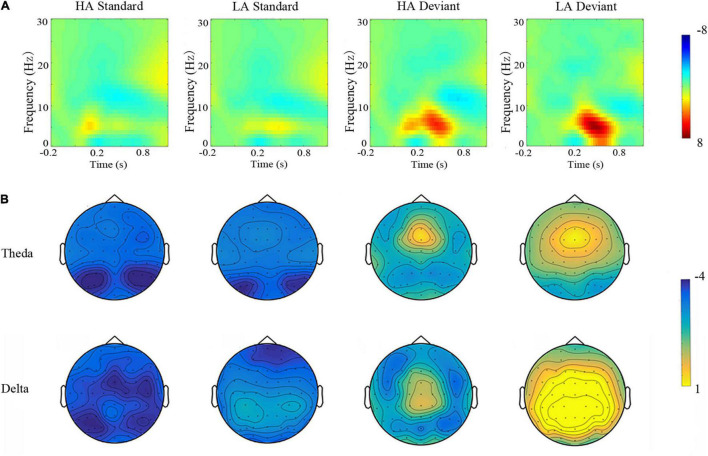
Top panel **(A)** time-frequency. The grand averaged short-time Fourier transform absolute power of delta (0.1–3 Hz) and theta (4–8 Hz) subcomponents in response to standard and deviant conditions for residents of LA and HA regions. Bottom panel **(B)** topographic maps. Scalp distributions of theta and delta band powers elicited by the standard and deviant conditions for LA and HA amplitude residents.

Theta band (4–8 Hz) measures (averaged across the nine electrode sites) demonstrated a significant main effect of Group [*F*(1, 69) = 9.936, *p* < 0.010, η^2^ = 0.135], with lower theta band power in the HA group. Stimulus type was also significant [*F*(1, 69) = 314.801, *p* < 0.001, η^2^ = 0.821], such that stronger theta band power was observed during the deviant condition (Figure 4). A significant Group × Stimulus Type interaction [*F*(1, 69) = 6,956, *p* < 0.05, η^2^ = 0.102] revealed that the lower theta band power observed in HA residents was only different from the LA group in deviant condition (i.e., no group differences for theta band power during standard conditions).

Delta band (0.1–3 Hz) power (averaged across the nine electrode sites) was also significantly different across Group [*F*(1,69) = 29.535, *p* < 0.001, η^2^ = 0.300], with weaker delta band power for HA residents, relative to the LA group. The main effect of Stimulus Type was significant [*F*(1,69) = 224.219, *p* < 0.001, η^2^ = 0.765], such that delta band power was stronger in the deviant condition and weaker in the standard condition (Figure 4). This was further qualified by a significant interaction [*F*(1,69) = 22.394, *p* < 0.050, η^2^ = 0.245] demonstrating that the HA group only elicited weaker delta band power (than the LA group) during the deviant condition; HA and LA groups did not differ in the standard condition (Table 4).

**TABLE 4:** Theta and delta band powers for standard and deviant conditions at F3, F4, Fz, FC3, FC4, FCz, C3, C4, and Cz sites in LA and HA groups (*M* ± *SD*).

	Standard		Deviant	
	Theta	Delta	Theta	Delta
HA	0.05 ± 0.79	−0.63 ± 0.80	2.67 ± 1.20	0.15 ± 1.15
LA	0.42 ± 0.97	1.39 ± 1.15	4.02 ± 2.05	4.02 ± 2.41

### Correlations

**FIGURE 5:**
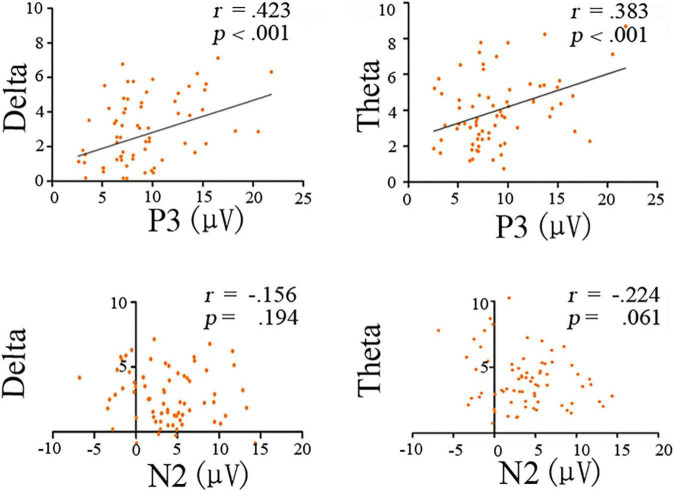
Scatterplot for correlations between time-frequency bands (theta and delta powers) and ERP mean amplitudes (N2 and P3) for the deviant condition.

The N2 ERP component was not significantly correlated with theta (*r* = −0.224, *p* = 0.061) or delta (*r* = −0.156, *p* = 0.196) band power (Figure 5). In contrast, significant positive correlations were found between P3 mean amplitudes and theta band power (*r* = 0.383, *p* = 0.001), as well as between P3 mean amplitude and delta band power (*r* = 0.423, *p* = 0.001; Figure 5).

## Discussion

This study aims to reveal the behavioral performance and neural processing of BIC after long-term exposure to HAs by immigrants from sea-level regions. A two-choice oddball paradigm with co-registered EEG records simultaneously evaluated BIC accuracy and RTs, the N2 and P3 ERP components, as well as theta and delta band powers. This research has crucial implications for people living in HA environments, such as impaired BIC, and difficulties inhibiting inappropriate behaviors for adaptive living can cause many issues for HA immigrants. Importantly, this research also provides a theoretical supplement for the clinical treatment of behavioral control disorder.

This study matches LA and HA groups on age, gender, intelligence, and education level, as these factors have been shown to influence BIC at the behavioral and neural levels (Fabiani and Friedman, 2010; Begum et al., 2011; Yuan et al., 2012). For instance, visual oddball tasks have been used to compare BIC in young and older adults, with results showing a stark decline in BIC performance for elderly adults (Fabiani and Friedman, 2010). Females have been found to perform faster and elicit stronger neural activity in response to deviant stimuli than do males (Yuan et al., 2012). Adults with higher levels of education are also faster than adults with lower levels of education for infrequent stimuli (Begum et al., 2011). Therefore, the current results highlight the significance of altitude in BIC, by demonstrating the persistence of this effect despite controlling for other demographic factors.

Behavioral results showed delayed RTs and lower accuracy scores for participants in the HA group than the LA group. These findings suggest that participants in the HA group may have needed to expend more time and effort in order to achieve their best performance scores but were still unable to match the behavioral performance of LA participants. Interestingly, delayed RTs of HA residents were seen not only for deviant stimuli but also for frequent, standard stimuli, indicating that individuals living in HA areas may have disrupted behavioral RTs in general. These results are consistent with previous studies demonstrating longer RTs on measures of attention, mental rotation, and memory for HA immigrants compared with sea-level residents (Bokura et al., 2001; Zhang et al., 2013, 2018a,b; Wang et al., 2015). From the behavioral view, it seems that long-term exposure (at least 3 years) to HAs influences cognitive abilities and RTs.

At the neuroimaging level, N2 and P3 amplitudes were maximal at frontocentral sites, consistent with these ERP components marking conflict monitoring and response inhibition indices of BIC in oddball paradigms (Liang et al., 2018; Ren et al., 2019). However, these components were significantly attenuated for participants in the HA group relative to individuals in the LA group (Bokura et al., 2001; Zhang et al., 2013, 2018a,b; Wang et al., 2015). Moreover, Group × Stimulus Type interactions did not reach significance. Therefore, N2 and P3 ERP responses were not differentially affected by altitude exposure across deviant and standard conditions. Recent ERP studies have implied that neural oscillations and synchronization show key mechanisms of interneuronal exchange (Luu and Tucker, 2001; Makeig et al., 2004; Ford et al., 2008; Roach and Mathalon, 2008). ERPs are voltage fluctuation at the averages of EEG events, and the “random” changing in the EEG cancels out, approaching zero as trial number augment. However, the time-frequency analysis of single-trial EEG epochs reveals that EEG does not only reflect random background noise. In fact, there are event-related changes in the magnitude and phase of EEG oscillations at specific frequencies that support their role in the processing of the event (Luu and Tucker, 2001; Makeig et al., 2004). Compared with ERPs, the time-frequency analysis may be more sensitive to the neuronal processes in cognitive abilities. For example, teams of Daniel have shown that both phase and power measures are more sensitive to schizophrenia than traditional ERP components such as the P300 (Ford et al., 2008).

The time-frequency analysis also has a strong relationship with components of the time-domain analysis, with delta and theta bands being the two most stable components associated with the P3 response (Polich, 2007; Ergen et al., 2008; Harper et al., 2014; Bachman and Bernat, 2018). The two separate, but highly overlapping, synergistic activations are sensitive to the demand for response inhibition during the P3 stage (Harper et al., 2014). Consistent with these results, this study unveils a significant correlation between P3 mean amplitudes and time-frequency bands in the theta and delta ranges. The power of theta and delta bands is also associated with response inhibition (Polich, 2007; Ergen et al., 2008; Harper et al., 2014; Papenberg et al., 2016; Bachman and Bernat, 2018). For instance, oddball paradigms have further revealed that the delta and theta band powers tend to be larger for low-frequency (deviant) stimuli than for high-frequency (standard) stimuli, once again reflecting response inhibition functions (Polich, 2007; Ergen et al., 2008; Bachman and Bernat, 2018). The present results are also in line with past research, indicating that theta and delta band powers are significantly enhanced over prefrontal regions during the presentation of low-frequent stimuli in both HA and LA groups (Polich, 2007; Ergen et al., 2008; Harper et al., 2014; Bachman and Bernat, 2018).

Previous study suggests that weaker theta and delta band powers represent compromised BIC constructs (Luu and Tucker, 2001; Makeig et al., 2004). For example, older adults demonstrate lower band powers over prefrontal areas in response to visually rare No-Go stimuli, accompanied by longer RTs and lower accuracies than young adults (De Blasio and Barry, 2018). This study extends our understanding of BIC further, by demonstrating modulations of theta and delta band powers across altitude groups. To be specific, theta and delta bands were reduced in the prefrontal regions in the HA group during deviant trials, although no differences were observed between LA and HA groups during standard trials. It stands to reason that long-term exposure to HAs might lead to BIC impairments in immigrants from LA regions, with a particular disruption of prefrontal lobe activity. Neuroimaging studies support the view that changes in the intensity of neural activity are influenced by the changes in the brain structure and/or neural metabolism (Barrós-Loscertales et al., 2006; Bélanger et al., 2011). Therefore, decreases in BIC for immigrants from HA areas are likely to be the result of structural brain changes and/or neural metabolism changes following long-term exposure to HAs. Decreased theta and delta band powers are associated with declines in prefrontal lobe gray matter volume (Barrós-Loscertales et al., 2006; Shah-Basak et al., 2019; Steiger et al., 2019), paralleling that seen for aging populations (Bernat et al., 2007; Taki et al., 2011; Steffener et al., 2013; Guo et al., 2014; Park et al., 2015; Pergher et al., 2019) and residents from HA regions (Liotti et al., 2000). These findings suggest that structural changes in gray matter volume and neural activity within the prefrontal lobe could be a possible explanation for the observed declines in BIC for individuals following long-term exposure to HAs. However, the EEG technique used, in this study, cannot speak to direct evidence of the structural change due to the poor spatial resolution of the method and the absence of pre- and post-structural measures. In this study, the prefrontal cortex is once again identified as the key region associated with BIC, with attenuated prefrontal lobe activation and weaker behavioral performance during deviant trials.

Oxygen is crucial to prime physical health, and with approximately 20% of oxygen intake being consumed by the brain, the brain is the most vulnerable organ to hypoxic exposure in HA environments (Bélanger et al., 2011; Wang et al., 2015; Papenberg et al., 2016). Cerebral energy metabolism can be affected by hypoxia (Watts et al., 2018), highlighting the close relationship between cerebral activity and oxygen consumption. In fact, task-dependent increases in cerebral activity are invariably accompanied by local blood flow and glucose utilization (Bélanger et al., 2011; Watts et al., 2018). The present results revealed weaker prefrontal activity in the HA group, and it appears that the deprived oxygen that supports in high amplitude regions can lead to alterations in neural metabolism rates and a subsequent decline of BIC prefrontal activation.

It is possible that HA exposure may have other potential influences on cognitive abilities and neural activation (e.g., atypical functional connectivity between subcortical and cortical networks). The present EEG results reveal the differences in BIC performance and prefrontal neural activity dependent on altitude exposures, although the contributions of subcortical areas related to BIC (e.g., thalamus and ACC; Schmaal et al., 2013; Liu et al., 2019) cannot be spoken directly in this study (due to the poor spatial resolution of EEG) but should be considered in future research. For example, EEG and fMRI techniques could be combined so as to simultaneously evaluate BIC neural mechanisms with both high temporal and spatial resolution.

## Conclusion

Sea-level immigrants that have been exposed to high amplitudes for a long period of time (at least 3 years) demonstrate attenuated ERP components marking behavioral inhibition (N2 and P3) relative to individuals residing in LA regions. Weaker prefrontal neural activity (power of theta and delta bands) and impaired behavioral performance were also observed for members of the HA group, indicating that chronic HA exposure can affect the processing speed and accuracy of BIC. These findings make a significant contribution to the furthering of our understanding of BIC mechanisms in relation to basic cognitive science from the perspective of altitude adaption.

## Data Availability Statement

The raw data supporting the conclusions of this article will be made available by the authors, without undue reservation.

## Ethics Statement

The studies involving human participants were reviewed and approved by Ethics Committee of Tibet University. The patients/participants provided their written informed consent to participate in this study. Written informed consent was obtained from the individual(s) for the publication of any potentially identifiable images or data included in this article.

## Author Contributions

JW, LZ, ZW, and XW performed the experiments. JW and LZ analyzed and interpreted the results. JW, XW, NM, TZ, KC, and BB drafted and revised the manuscript. All authors contributed to the design of the experiments, read, and approved the final manuscript.

## Conflict of Interest

The authors declare that the research was conducted in the absence of any commercial or financial relationships that could be construed as a potential conflict of interest.

## Publisher’s Note

All claims expressed in this article are solely those of the authors and do not necessarily represent those of their affiliated organizations, or those of the publisher, the editors and the reviewers. Any product that may be evaluated in this article, or claim that may be made by its manufacturer, is not guaranteed or endorsed by the publisher.

## References

[B1] Ames S. L. Wong S. W. Bechara A. (2014). Neural correlates of a Go/NoGo task with alcohol stimuli in light and heavy young drinkers. *Behav. Brain Res.* 274 382–389. 10.1016/j.bbr.2014.08.039 25172182 PMC4179865

[B2] Anguera J. A. Boccanfuso J. Rintoul J. L. (2013). Video game training enhances cognitive control in older adults. *Nature* 501 97–101. 24005416 10.1038/nature12486 PMC3983066

[B3] Bachman M. D. Bernat E. M. (2018). Independent contributions of theta and delta time-frequency activity to the visual oddball P3b. *Int. J. Psychophysiol.* 128 70–80. 10.1016/j.ijpsycho.2018.03.010 29574233 PMC5960636

[B4] Barrós-Loscertales A. Meseguer V. Sanjuán A. Belloch V. Parcet M. A. Torrubia R. (2006). Behavioral inhibition system activity is associated with increased amygdala and hippocampal gray matter volume: a voxel-based morphometry study. *Neuroimage* 33 1011–1015. 10.1016/j.neuroimage.2006.07.025 16979909

[B5] Begum T. Reza F. Ahmed A. L. Elaina S. Abdullah J. M. (2011). “Delta signal in high educational level in auditory oddball paradigm-a Wavelet study,” in *Proceedings of the 2011 4th International Congress on Image and Signal Processing* (Shanghai: IEEE).

[B6] Bélanger M. Allaman I. Magistretti P. J. (2011). Brain energy metabolism: focus on astrocyte-neuron metabolic cooperation. *Cell Metab.* 14 724–738. 10.1016/j.cmet.2011.08.016 22152301

[B7] Bernat E. M. Malone S. M. Williams W. J. Patrick C. J. Iacono W. G. (2007). Decomposing delta, theta, and alpha time–frequency erp activity from a visual oddball task using pca. *Int. J. Psychophysiol.* 64 62–74. 10.1016/j.ijpsycho.2006.07.015 17027110 PMC2276568

[B8] Bokura H. Yamaguchi S. Kobayashi S. (2001). Electrophysiological correlates for response inhibition in a Go/NoGo task. *Clin. Neurophysiol.* 112 2224–2232. 10.1016/s1388-2457(01)00691-5 11738192

[B9] Bucuk M. Tomic Z. TuskanMohar L. (2008). Recurrent transient global amnesia at high altitude. *High Alt. Med. Biol.* 9 239–240. 10.1089/ham.2008.0002 18800962

[B10] Carlson S. M. (2005). Developmentally sensitive measures of executive function in preschool children. *Dev. Neuropsychol.* 28 595–616. 10.1207/s15326942dn2802_3 16144429

[B11] Carlson S. M. Moses L. J. (2001). Individual differences in inhibitory control and children’s theory of mind. *Child Dev.* 72 1032–1053. 10.1111/1467-8624.00333 11480933

[B12] Cavanagh J. F. Cohen M. X. (2009). Prelude to and resolution of an error: EEG phase synchrony reveals cognitive control dynamics during action monitoring. *J. Neurosci.* 29 98–105. 10.1523/JNEUROSCI.4137-08.2009 19129388 PMC2742325

[B13] Cavanagh J. F. Frank M. J. Klein J. (2010). Theresa, frontal theta links prediction errors to behavioral adaptation in reinforcement learning. *Neuroimage* 49 3198–3209. 10.1016/j.neuroimage.2009.11.080 19969093 PMC2818688

[B14] Chen A. Xu P. Wang Q. (2008). The timing of cognitive control in partially incongruent categorization. *Hum. Brain Mapp.* 29 1028–1039. 10.1002/hbm.20449 17894393 PMC6871019

[B15] Cooper P. S. Hughes M. E. (2017). Impaired theta and alpha oscillations underlying stopsignal response inhibition deficits in schizophrenia. *Schizophr. Res.* 193 474–476. 10.1016/j.schres.2017.08.002 28797527

[B16] De Blasio F. M. Barry R. J. (2018). Prestimulus delta and theta contributions to equiprobable Go/NoGo processing in healthy ageing. *Int. J. Psychophysiol.* 130 40–52. 10.1016/j.ijpsycho.2018.05.005 29775640

[B17] Delorme A. Makeig S. (2004). EEGLAB: an open source toolbox for analysis of single-trial EEG dynamics including independent component analysis. *J. Neurosci. Methods* 134 9–21. 10.1016/j.jneumeth.2003.10.009 15102499

[B18] Delorme A. Sejnowski T. Makeig S. (2007). Enhanced detection of artifacts in EEG data using higher-order statistics and independent component analysis. *Neuroimage* 34 1443–1449. 10.1016/j.neuroimage.2006.11.004 17188898 PMC2895624

[B19] Ergen M. Marbach S. Brand A. Başar-Eroğlu C. Demiralp T. (2008). P3 and delta band responses in visual oddball paradigm in schizophrenia. *Neurosci. Lett.* 440 304–308. 10.1016/j.neulet.2008.05.054 18571323

[B20] Fabiani M. Friedman D. (2010). Changes in brain activity patterns in aging: the novelty oddball. *Psychophysiology* 32 579–594. 10.1111/j.1469-8986.1995.tb01234.x 8524992

[B21] Ford J. M. Roach B. J. Hoffman R. S. Mathalon D. H. (2008). The dependence of P300 amplitude on gamma synchrony breaks down in schizophrenia. *Brain Res.* 1235 133–142. 10.1016/j.brainres.2008.06.048 18621027 PMC3230270

[B22] Goldstein M. Brendel G. Tuescher O. (2007). Neural substrates of the interaction of emotional stimulus processing and motor inhibitory control: an emotional linguistic go/no-go fMRI study. *Neuroimage* 36 1026–1040. 10.1016/j.neuroimage.2007.01.056 17509899

[B23] Guo Y. Zhang Z. Zhou B. Wang P. Yao H. Yuan M. (2014). Grey-matter volume as a potential feature for the classification of Alzheimer’s disease and mild cognitive impairment: an exploratory study. *Neurosci. Bull.* 30 477–489. 10.1007/s12264-013-1432-x 24760581 PMC5562611

[B24] Hailin M. Xiaoyan L. Ming L. Huifang M. Delong Z. (2018). Mental rotation effect on adult immigrants with long-term exposure to high altitude in Tibet: an ERP study. *Neuroscience* 386 339–350. 30049664 10.1016/j.neuroscience.2018.06.038

[B25] Harper J. Malone S. M. Bernat E. M. (2014). Theta and delta band activity explain n2 and P3 ERP component activity in a go/no-go task. *Clin. Neurophysiol.* 125 124–132. 10.1016/j.clinph.2013.06.025 23891195 PMC4312493

[B26] Liang Q. Lin J. Yang J. Li X. Chen Y. Meng X. (2018). Intervention effect of repetitive TMS on behavioral adjustment after error commission in long-term methamphetamine addicts: evidence from a two-choice oddball task. *Neurosci. Bull.* 34 449–456. 10.1007/s12264-018-0205-y 29340869 PMC5960444

[B27] Liddle P. F. Kiehl K. A. Smith A. M. (2001). Event-related fMRI study of response inhibition. *Hum. Brain Mapp.* 12 100–109. 11169874 10.1002/1097-0193(200102)12:2<100::AID-HBM1007>3.0.CO;2-6 PMC6871906

[B28] Liotti M. Woldorff M. G. Perez R. Mayberg H. S. (2000). An ERP study of the temporal course of the Stroop color-word interference effect. *Neuropsychologia* 38 701–711. 10.1016/s0028-3932(99)00106-2 10689046

[B29] Liu N. Tong X. Huang W. Fu J. Xue X. (2019). Synaptic injury in the thalamus accompanies white matter injury in hypoxia/ischemia-mediated brain injury in neonatal rats. *Biomed. Res. Int.* 2019 1–10. 10.1155/2019/5249675 PMC6803747 31687391

[B30] Luu P. Tucker D. M. (2001). Regulating action: alternating activation of midline frontal and motor cortical networks. *Clin. Neurophysiol.* 112 1295–1306. 10.1016/s1388-2457(01)00559-4 11516742

[B31] Ma H. Zhang D. Li X. Ma H. Wang N. Wang Y. (2019). Long-term exposure to high altitude attenuates verbal and spatial working memory: evidence from an event-related potential study. *Brain Behav.* 9:e01256. 10.1002/brb3.1256 30891949 PMC6456776

[B32] Makeig S. Debener S. Onton J. Delorme A. (2004). Mining eventrelated brain dynamics. *Trends Cogn. Sci.* 8 204–210. 15120678 10.1016/j.tics.2004.03.008

[B33] Morein-Zamir S. Dodds C. van Hartevelt T. J. (2014). Hypoactivation in right inferior frontal cortex is specifically associated with motor response inhibition in adult ADHD. *Hum. Brain Mapp.* 35 5141–5152. 10.1002/hbm.22539 24819224 PMC4336557

[B34] Oostenveld R. Fries P. Maris E. Schoffelen J. M. (2011). FieldTrip: open source software for advanced analysis of MEG, EEG, and invasive electrophysiological data. *Comput. Intell. Neurosci.* 2011:156869. 10.1155/2011/156869 21253357 PMC3021840

[B35] Papenberg G. Ferencz B. Mangialasche F. Mecocci P. Cecchetti R. Kalpouzos G. (2016). Physical activity and inflammation: effects on gray-matter volume and cognitive decline in aging. *Hum. Brain Mapp.* 37 3462–3473. 10.1002/hbm.23252 27159568 PMC6867433

[B36] Park S. H. Kim H. Lee K. J. (2015). Correlations between homocysteine and grey matter volume in patients with Alzheimer’s disease. *Psychogeriatrics* 15 116–122. 10.1111/psyg.12082 25560091

[B37] Penaloza D. Arias-Stella J. (2007). The heart and pulmonary circulation at high altitudes: healthy highlanders and chronic mountain sickness. *Circulation* 115 1132–1146. 10.1161/circulationaha.106.624544 17339571

[B38] Pergher V. Tournoy J. Schoenmakers B. Van Hulle M. M. (2019). 300, Gray matter volume and individual characteristics correlates in healthy elderly. *Front. Aging Neurosci.* 11:104. 10.3389/fnagi.2019.00104 31130855 PMC6510164

[B39] Polich J. (2007). Updating P300: an integrative theory of P3a and P3b. *Clin. Neurophysiol.* 118 2128–2148. 10.1016/j.clinph.2007.04.019 17573239 PMC2715154

[B40] Prisciandaro J. J. Joseph J. E. Myrick H. McRae-Clark A. L. Henderson S. Pfeifer J. (2014). The relationship between years of cocaine use and brain activation to cocaine and response inhibition cues. *Addiction* 109 2062–2070. 10.1111/add.12666 24938849 PMC4229403

[B41] Ren Z. Yang J. Yuan J. (2019). Unconscious impulsivity control maintains the ability of behavioral inhibitory control in males: evidence of reaction-time cost. *Psych. J.* 8 330–341. 10.1002/pchj.299 31230424

[B42] Roach B. J. Mathalon D. H. (2008). Event-related EEG time-frequency analysis: an overview of measures and an analysis of early gamma band phase locking in schizophrenia. *Schizophr. Bull.* 34:907. 10.1093/schbul/sbn093 18684772 PMC2632478

[B43] Roberts L. E. Rau H. Lutzenberger W. Birbaumer N. (1994). Mapping P300 waves onto inhibition: Go/No-Go discrimination. *Electroencephalogr. Clin. Neurophysiol.* 92 44–55. 10.1016/0168-5597(94)90006-x 7508852

[B44] Schmaal L. Joos L. Koeleman M. Veltman D. J. Wim V. D. B. Goudriaan A. E. (2013). Effects of modafinil on neural correlates of response inhibition in alcohol-dependent patients. *Biol. Psychiatry* 73 211–218. 10.1016/j.biopsych.2012.06.032 22858150

[B45] Sehlmeyer C. Konrad C. Zwitserlood P. Arolt V. Falkenstein M. Beste C. (2010). ERP indices for response inhibition are related to anxiety-related personality traits. *Neuropsychologia* 48 2488–2495. 10.1016/j.neuropsychologia.2010.04.022 20434466

[B46] Shah-Basak P. P. Kielar A. Deschamps T. Verhoeff N. P. Jokel R. Meltzer J. (2019). Spontaneous oscillatory markers of cognitive status in two forms of dementia. *Hum. Brain Mapp.* 40 1594–1607. 10.1002/hbm.24470 30421472 PMC6865664

[B47] Simmonds D. J. Pekar J. J. Mostofsky S. H. (2008). Meta-analysis of Go/No-go tasks demonstrating that fMRI activation associated with response inhibition is task-dependent. *Neuropsychologia* 46 224–232. 10.1016/j.neuropsychologia.2007.07.015 17850833 PMC2327217

[B48] Singh S. B. Thakur L. Anand J. P. (2003). Effect of high altitude (HA) on event related brain potentials. *Indian J. Physiol. Pharmacol.* 47 52–58. 12708124

[B49] Stadler C. Sterzer P. Schmeck K. (2007). Reduced anterior cingulate activation in aggressive children and adolescents during affective stimulation: association with temperament traits. *J. Psychiatr. Res.* 41 410–417. 10.1016/j.jpsychires.2006.01.006 16516233

[B50] Steffener J. Brickman A. M. Habeck C. G. Salthouse T. A. Stern Y. (2013). Cerebral blood flow and gray matter volume covariance patterns of cognition in aging. *Hum. Brain Mapp.* 34 3267–3279. 10.1002/hbm.22142 22806997 PMC3812339

[B51] Steiger T. K. Herweg N. A. Menz M. M. Bunzeck N. (2019). Working memory performance in the elderly relates to theta-alpha oscillations and is predicted by parahippocampal and striatal integrity. *Sci. Rep.* 9:706. 10.1038/s41598-018-36793-3 30679512 PMC6345832

[B52] Taki Y. Kinomura S. Sato K. Goto R. Kawashima R. Fukuda H. (2011). A longitudinal study of gray matter volume decline with age and modifying factors. *Neurobiol. Aging* 32 907–915. 10.1016/j.neurobiolaging.2009.05.003 19497638

[B53] Virués-Ortega J. Garrido E. Javierre C. Kloezeman K. C. (2006). Human behaviour and development under high-altitude conditions. *Dev. Sci.* 9 400–410. 10.1111/j.1467-7687.2006.00505.x 16764613

[B54] Wang Y. Ma H. L. Fu S. M. (2015). Long-term exposure to high altitude affects voluntary spatial attention at early and late processing stages. *Sci. Rep.* 4:4443. 10.1038/srep04443

[B55] Wang Y. Yang J. Yuan J. (2011). The impact of emotion valence on brain processing of behavioral inhibitory control: spatiotemporal dynamics. *Neurosci. Lett.* 502 112–116. 10.1016/j.neulet.2011.07.039 21827832

[B56] Watts M. E. Pocock R. Claudianos C. (2018). Brain energy and oxygen metabolism: emerging role in normal function and disease. *Front. Mol. Neurosci.* 22:216. 10.3389/fnmol.2018.00216 PMC6023993 29988368

[B57] Womelsdorf T. Johnston K. Vinck M. (2010). Theta-activity in anterior cingulate cortex predicts task rules and their adjustments following errors. *Proc. Natl. Acad. Sci. U.S.A.* 107 5248–5253. 10.1073/pnas.0906194107 20194767 PMC2841867

[B58] Yan X. Zhang J. Gong Q. (2011a). Adaptive influence of long term high altitude residence on spatial working memory: an fMRI study. *Brain Cogn.* 77 53–59. 10.1016/j.bandc.2011.06.002 21767899

[B59] Yan X. Zhang J. Gong Q. (2011b). Prolonged high-altitude residence impacts verbal working memory: an fMRI study. *Exp. Brain Res.* 208 437–445. 10.1007/s00221-010-2494-x 21107542

[B60] Yuan J. He Y. Qinglin Z. (2008). Gender differences in behavioral inhibitory control: ERP evidence from a two-choice oddball task. *Psychophysiology* 45 986–993. 10.1111/j.1469-8986.2008.00693.x 18778319

[B61] Yuan J. Meng X. Yang J. (2012). The valence strength of unpleasant emotion modulates brain processing of behavioral inhibitory control: neural correlates. *Biol. Psychol.* 89 240–251. 10.1016/j.biopsycho.2011.10.015 22056697

[B62] Yuan J. J. Liu W. J. Liang Q. D. Cao X. Y. Lucas M. V. Yuan T. F. (2020). Effect of low-frequency repetitive transcranial magnetic stimulation on impulse inhibition in abstinent patients with methamphetamine addiction a randomized clinical trial. *JAMA Netw. Open* 3:e200910. 10.1001/jamanetworkopen.2020.0910 32167568 PMC7070234

[B63] Zhang D. Ma H. Huang J. Zhang X. Ma H. Liu M. (2018a). Exploring the impact of chronic high-altitude exposure on visual spatial attention using the ERP approach. *Brain Behav.* 8:e00944. 10.1002/brb3.944 29761004 PMC5943834

[B64] Zhang D. Zhang X. Ma H. Wang Y. Ma H. Liu M. (2018b). Competition among the attentional networks due to resource reduction in tibetan indigenous residents: evidence from event-related potentials. *Sci. Rep.* 8:610. 10.1038/s41598-017-18886-7 29330442 PMC5766594

[B65] Zhang J. Zhang H. Li J. (2013). Adaptive modulation of adult brain gray and white matter to high altitude: structural MRI studies. *PLoS One* 8:e68621. 10.1371/journal.pone.0068621 23874692 PMC3712920

